# Diabetes Patient Surveillance in the Emergency Department: Proof of Concept and Opportunities

**DOI:** 10.5811/westjem.2020.12.49171

**Published:** 2021-04-02

**Authors:** M. Gabriela Sava, Ronald G. Pirrallo, Brian C. Helsel, Jingyuan Tian, Patricia Carbajales-Dale, Kuang-Ching Wang, John Bruch, Ronald W. Gimbel

**Affiliations:** * Clemson University, Department of Management, Clemson, South Carolina; † University of South Carolina School of Medicine Greenville, Department of Emergency Medicine, Greenville, South Carolina; ‡ University of Kansas Medical Center, Department of Internal Medicine, Kansas City, Kansas; § Clemson University, Center for Geospatial Technologies, Clemson, South Carolina; ¶ University of South Carolina School of Medicine Greenville, Department of Internal Medicine, Greenville, South Carolina; || Clemson University, Department of Public Health Sciences, Clemson, South Carolina; # Clemson University, Department of Electrical and Computer Engineering, Clemson, South Carolina

## Abstract

**Introduction:**

The purpose of this study was to characterize the at-risk diabetes and prediabetes patient population visiting emergency department (ED) and urgent care (UC) centers in upstate South Carolina.

**Methods:**

We conducted this retrospective study at the largest non-profit healthcare system in South Carolina, using electronic health record (EHR) data of patients who had an ED or UC visit between February 2, 2016–July 31, 2018. Key variables including International Classification of Diseases, 10th Revision codes, laboratory test results, family history, medication, and demographic characteristics were used to classify the patients as healthy, having prediabetes, having diabetes, being at-risk for prediabetes, or being at-risk for diabetes. Patients who were known to have diabetes were classified further as having controlled diabetes, management challenged, or uncontrolled diabetes. Population analysis was stratified by the patient’s annual number of ED/UC visits.

**Results:**

The risk stratification revealed 4.58% unique patients with unrecognized diabetes and 10.34% of the known patients with diabetes considered to be suboptimally controlled. Patients identified as diabetes management challenged had more ED/UC visits. Of note, 33.95% of the patients had unrecognized prediabetes/diabetes risk factors identified during their ED/UC with 87.95% having some form of healthcare insurance.

**Conclusion:**

This study supports the idea that a single ED/UC unscheduled visit can identify individuals with unrecognized diabetes and an at-risk prediabetes population using EHR data. A patient’s ED/UC visit, regardless of their primary reason for seeking care, may be an opportunity to provide early identification and diabetes disease management enrollment to augment the medical care of our community.

## INTRODUCTION

Evidence continues to support preventive services as one solution to reducing patient morbidity and mortality and decreasing healthcare system demands and costs.^1^,^2^ Appropriately, healthcare system decision-makers have shifted their focus toward preventive screening, early detection, and management of chronic diseases such as diabetes. The US Centers for Disease Control and Prevention (CDC) reports 30.3 million Americans (9.4%) have diabetes and another 84.1 million (33.9%) have prediabetes.^3^ South Carolina ranks seventh highest in the nation for an adult population with diabetes, and approximately 1 in 6 African-Americans living in South Carolina have diabetes.^4^ In addition, the emergency department (ED) and urgent care (UC) patient population is known to have a high prevalence of diabetes risk factors and undiagnosed diabetes.^5^ As an episodic and unscheduled access point into the US healthcare system, an ED or UC visit is an ideal location for acute disease management and public health surveillance of a community’s burden with diabetes.

Prior research demonstrates that ED diabetes surveillance protocols using only random, blood glucose measurements have been successful.^6^–^10^ Patient characteristics, such as demographics,^11^ body mass index (BMI), family history, comorbidities, and laboratory measures of impaired glucose tolerance, hemoglobin A1c (HbA1c), cholesterol, and triglycerides, can be used to refine a screening decision for diabetes. Confirmatory testing such as fasting plasma glucose, oral glucose tolerance testing, or HbA1C should be considered for definitive diabetes diagnosis.

The adoption of a healthcare systemwide, patient electronic health record (EHR) makes it possible to use dynamic and continuous patient data inquiry for real-time clinical decision-making. Decision-making heuristics and algorithms are being advanced to help notify and advise clinicians of an at-risk patient. In addition, as ED clinicians continue to expand their scope of practice toward early detection and morbidity reduction, exampled by successful human immunodeficiency virus^12^ and opioid-misuse screening,^13^ understanding the characteristics of an at-risk population is paramount.

In a proof-of-concept data exploration and risk classification study, we sought to describe the at-risk population for prediabetes and diabetes and those with suboptimally controlled diabetes in the general ED and UC population in upstate South Carolina. Using clinical classification rules based on variables commonly collected in a patient’s EHR and the American Diabetes Association (ADA) Standards of Medical Care in Diabetes,^14^ we described the characteristics of the broad at-risk diabetes patient population that may not otherwise interact with the healthcare system. Additionally, we geographically mapped the at-risk population to reveal where resources such as primary care clinics or chronic disease management programs access should be focused and allocated.

## METHODS

This was a retrospective descriptive study of a single healthcare system’s EHR containing data on patients who presented to the ED or UC centers of the study location, a health system in South Carolina between February 2, 2016–July 31, 2018. The health system provides comprehensive healthcare for the 11 counties in upstate South Carolina serving a population of 1.4 million and is the region’s largest health system. The study location’s department of emergency medicine includes seven hospital-based EDs and six UC centers. The hospital-based EDs range from rural access hospitals to an academic American College of Surgeons-verified Level I trauma center that, in total, serve more than 360,000 emergent patients annually. The six UC centers are open 16 hours each weekday with 14 hours of weekend hourly coverage seeing approximately 100,000 patients annually. The health system’s institutional review board determined this study not to constitute human subjects research.

**Population Health Research Capsule**

What do we already know about this issue?

*Prior research demonstrates that emergency department (ED) diabetes surveillance protocols using random blood glucose measurements have been successful.*

What was the research question?

*Our goal was to characterize the at-risk diabetes and prediabetes patient population visiting ED and urgent care (UC) centers in upstate South Carolina.*

What was the major finding of the study?

*A single ED/UC unscheduled visit can identify individuals with unrecognized diabetes and an at-risk prediabetes using electronic health records data.*

How does this improve population health?

*A patient’s ED/UC visit, independent of the reason, may be an opportunity to provide early identification and diabetes disease management enrollment.*

### Study Population

For all patients 18 years of age and older, their ED and UC visits were considered a sentinel event to query diabetes-specific screening variables included in the EHR. For patients with multiple visits, only the most recent visit was considered for their risk classification and labeling. Data were extracted from the EHR based on a predetermined set of variables selected by the researchers. These variables contained a preset data code that healthcare system report writers aggregated to generate the final subject dataset for analysis. All reports generated by the report writers were merged using a patient identifier as a linking pin and then de-identified for our analysis. The healthcare system report writers were blinded to the study purpose and hypothesis development.

The anonymized data used to define the risk classification included the following: a) patient demographics; b) insurance status; c) ED/UC visit acquired or previously entered laboratory results: glucose, HbA1C, triglycerides, high-density lipoprotein cholesterol; (d) presence of 12 classes of diabetes-related medication, oral or injectable; (e) problem list, entered by the healthcare providers; (f) self-reported diabetes-related family history; and g) diabetes-related diagnosis and International Classification of Diseases, 10^th^ Revision (ICD-10) codes. We performed data processing and classification using Microsoft Excel 2016 (Microsoft Corporation, Redmond, WA) and Stata package v 14.2 (StataCorp, College Station, TX).

**Figure 1:**
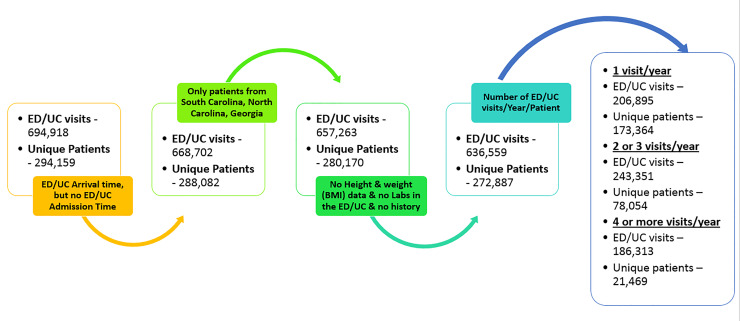
Data processing elimination rules and final sub-populations creation. *ED*, emergency department; *UC*, urgent care; *BMI*, body mass index.

Figure 1 describes the data processing elimination rules used to identify the patient subpopulations with one, two or three, or more than four ED/UC visits/year. Patients with incomplete information regarding ED/UC admission date, BMI, laboratory results, past medical history, and patients who resided in other states than South Carolina, North Carolina, and Georgia were sequentially eliminated from the original data pool. To further homogenize the patient population for analysis, we grouped patients into one of three categories based on their total number of ED visits within our time horizon. These subpopulations were used to further define the risk classification of the patients and to explore the differences as a function of ED/UC utilization.

### Risk Classification Rules

**Figure 2:**
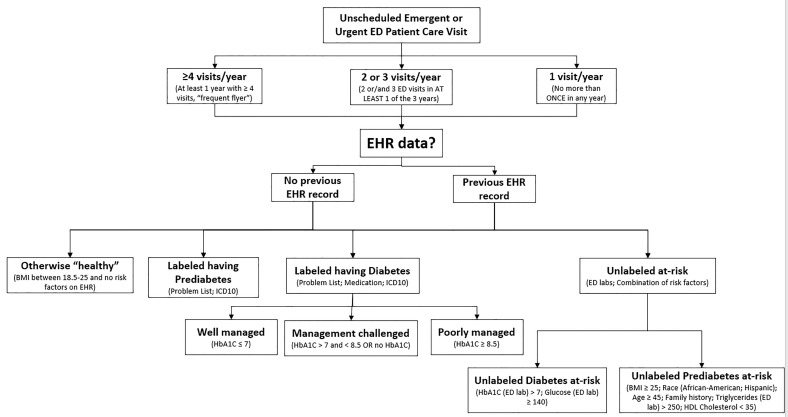
Decision-making process for patients’ classification. *ED*, emergency department; *EHR*, electronic health records; *BMI*, body mass index; *HbA1C*, hemoglobin A1C; *HDL*, high-density lipoprotein.

Using only EHR information, we classified each patient based on modified ADA screening guidelines^14^ and the study location’s definitions for diabetes chronic disease management. Previous diabetes diagnosis, ICD-10 diabetes-related codes, diabetes-related problems on the patient problem list, family history, past laboratory values, or hypoglycemic medications were all considered equivalent for labeling purposes. We determined final patient risk classifications using the decision-making process presented in Figure 2.

The four main classes of patients and their classification rules included the following:

1. *Otherwise “healthy”*: no prediabetes or diabetes diagnosis or characteristics that indicate a negligible risk of acquiring the disease.
  Classification rules:
  IF patient has BMI < 25 and NO risk factors as per the ADA screening guidelines.^14^
2. *Labeled having prediabetes*: diagnosis present.
  Classification rules:
  IF patient has (1) the ICD-10 code R73 present *OR* (2) problem list indicates the diagnosis.
3. *Labeled having diabetes*: diagnosis present and disease management recorded.
  Classification rules:
  IF patient has (1) one of the ICD-10 codes E08, E09, E10, E11, E13, O24 present *OR* (2) problem list indicates the diagnosis *OR* (3) diabetes medication prescribed, oral or injectable.
  3.1. *Well managed*: HbA1C value present and ≤ 7%.
    Classification rules:
    IF HbA1C test value ≤ 7% (1) during the ED/UC visit *OR* (2) from the EHR.
  3.2. *Management challenged*: no HbA1C value or value between 7% and 8.5%.
    Classification rules:
    IF the patient HbA1C test value is between 7% and 8.5% (1) during the ED/UC visit *OR* (2) from the EHR *OR* (3) no record of the test exists.
  3.3. *Poorly managed*: HbA1c ≥ 8.5%.
    Classification rules:
    IF the patient HbA1C test value ≥ 8.5% (1) during the ED/UC visit *OR* (2) from the EHR.
4. *Unlabeled at-risk*: undiagnosed prediabetes or diabetes with at-risk characteristics.
  4.1. *Unlabeled diabetes at-risk*: tests values during the ED/UC visit outside the normal range.
    Classification rules:
    IF the patient (1) HbA1C test value > 7% during the ED/UC visit *OR* (2) glucose test value ≥ 140 milligrams per deciliter (mg/dL).
  4.2. *Unlabeled prediabetes at risk*: combination of diabetes risk factors as per ADA screening guidelines.^11^
    Classification rules:
    IF the patient satisfies one of the following: (1) BMI ≥ 25 and race – African-American *OR* (2) BMI ≥ 25 and race – Hispanic *OR* (3) BMI ≥ 25 and Age ≥ 45 *OR* (4) BMI ≥ 25 and family history indicates: diabetes, diabetes type I, diabetes type II, gestational diabetes, diabetic kidney disease or metabolic syndrome *OR* (5) BMI ≥ 25 and triglycerides ≥ 25 mg/dL test values from ED/UC or EHR *OR* (6) BMI ≥ 25 and HDL cholesterol < 35 mg/dL test values from ED/UC.
    IF a patient has no BMI, but one of the following combinations of risk factors: (1) race – African-American and age ≥ 45 *OR* (2) race – African-American and family history indicates diabetes-related diagnosis *OR* (3) race – African-American and triglycerides ≥ 250 mg/dL test values from ED/UC or HER *OR* (4) race – African-American and HDL cholesterol < 35 mg/dL test values from ED/UC *OR* (5) race – Hispanic and age ≥ 45 *OR* (6) race – Hispanic and family history indicates diabetes-related diagnosis *OR* (7) race – Hispanic and triglycerides ≥ 250 mg/dL test values from ED/UC or EHR *OR* (8) race – Hispanic and HDL cholesterol < 35 mg/dL test values from ED/UC.

### Testing the Risk Classification Results Against the National Averages

Based on the classification described in Figure 2, we classified the proportion of patients captured by each category of interest. A post hoc test of appropriateness of the ED/UC sample data comparing the subpopulation of diabetes prevalence to national averages included a Z-test statistic. Nationally 7.17% of the population has diabetes, 2.23% have undiagnosed diabetes, and 33.90% are individuals with prediabetes.^3^

### Risk Classification Mapping for Upstate South Carolina At-risk Population

For mapping purposes, the ZIP codes used were self-reported by the patients during their ED/UC visit. No verification of the address was made to attest whether the patient resided at that address. We removed ZIP codes that corresponded to post office boxes, specific companies, or organizations. Patient data were then geocoded using ZIP code boundaries defined by the US Postal Service for 2018 and compiled by TomTom (TomTom International N.V., Amsterdam, Netherlands) in Esri format (Environmental Systems Research Institute, Redlands, CA).^15^ We calculated prevalence for each category by dividing the number of patients by estimated total population in that ZIP code for 2018. Population estimates were obtained from 2019–2024 Esri updated demographics.^16^ Once calculated, prevalence rates for each ZIP code in upstate South Carolina were represented using equal interval or natural breaks classification function of the distribution of the data. All maps presented were obtained using Esri’s ArcGIS software. Further, we analyzed the prevalence of labeled and unlabeled patients with diabetes as a function of race/ethnic background, which is known to be an important discriminating factor.^11^

## RESULTS

Using the classification process described in Figure 2 and the risk classification rules, the following summary risk classification was obtained for each of the three subpopulations of interest (Table 1) and race/ethnic backgrounds. (Table 2).

The Z-test statistic that compared the sample proportions to the national averages for all three subpopulations were significant (*P*-values < 0.05): labeled having diabetes, 95% confidence interval [CI], 10.62%–10.85%; unlabeled diabetes at-risk, 95% CI, 4.50%–4.66%; labeled having prediabetes, 95% CI, 34.29%–34.65%. Thus, the proportion of disease identified in the sample, for each category, is greater in upstate South Carolina than the reported national levels.

**Figure 3:**
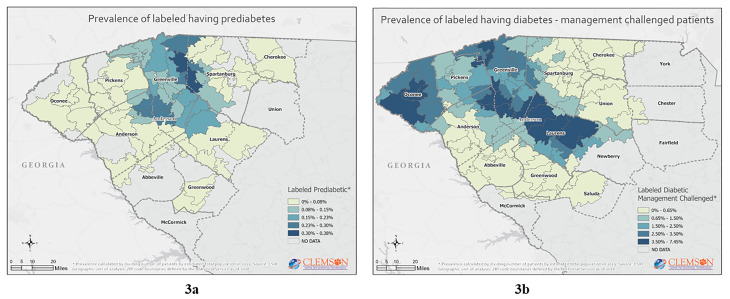

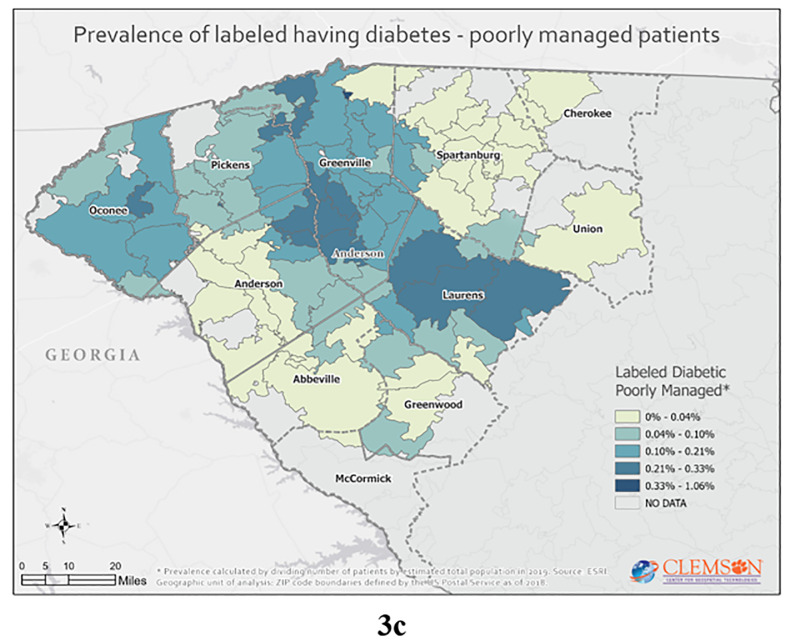
Labeled prediabetes or diabetes diagnosis: (3a) labeled having prediabetes; (3b) labeled having diabetes – management challenged; (3c) labeled having diabetes – poorly managed.

**Figure 4:**
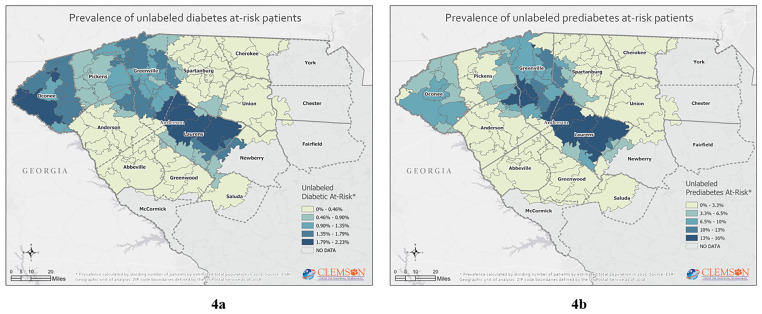
Unlabeled prediabetes or diabetes: (4a) unlabeled diabetes at risk; (4b) unlabeled prediabetes at risk.

The mapping further identified the areas of highest prevalence of our at-risk population of interest, Figures 3 and 4.

## DISCUSSION

This proof-of-concept study supports the idea that an ED/UC unscheduled visit can identify individuals with diabetes and at risk for diabetes in the population using EHR data. Our risk stratification revealed 4.58% unique patients with unrecognized diabetes, with 10.34% of the known individuals with diabetes considered to be suboptimally controlled. As expected, the patients posing diabetes management challenges had more ED/UC visits. Yet the percentage of unrecognized individuals with diabetes was similar across the patients with 1–3 or more ED/UC visits per year, around 4%. In addition, 33.95% of the patients had prediabetes risk factors identified during their ED/UC visit.

The prevalence of diabetes is known to be related to race/ethinicity^11^ of the population. Our data sample from upstate South Carolina demonstrates a disproportionate prevalence in the race categories, with 74.68% of our patients being White.^11^ And while our data are from an undifferentiated population that includes healthy and at-risk diabetes patients, our prevalence results of the disease identify similar race/ethnicity disparities compared to the national level. For example, our sample included 4.51% Hispanic, of whom 8.37% were labeled having diabetes and 5.15% were unlabeled patients at risk for diabetes. Our results may reflect other geographic and cultural characteristic present in South Carolina.

A patient’s ED or UC visit, regardless of their primary reason for seeking care, may be an opportunity to provide early identification and disease management enrollment to augment the healthcare safety net of the community. Collaboration with and referral to chronic disease management programs may be facilitated with the recognition that most of the patients in this community have some form of insurance, 87.95%.

When comparing the proportions obtained in the ED/UC sample data vs the national averages, we observed that indeed the sample proportions are higher. Even though the national averages tend to underestimate the disease prevalence for the areas with increased number of cases, the clinical definitions used to label our patients are more conservative than the ones used to generate the national averages.^3^ Thus, our estimate is conservative.

Not surprising, the mapping suggests that social determinants of health may influence where the at-risk prediabetes and diabetes population resides. Linkages with our dataset to other public health surveillance, economic, educational, and demographic data sources may further inform decision-makers on the best interventions to pursue.^17^ Our data suggest no single demographic-, geographic- or socioeconomic-focused intervention will likely be successful to reduce diabetes prevalence in upstate South Carolina.

The automated identification by the EHR system of an at-risk patient, based on his/her characteristics, could inform the healthcare provider to start an early detection or diseases management improvement process for that individual patient. The future ED/UC role may include identification of the at-risk patients who could benefit from an unscheduled preventive screening for diabetes, ordering a screening HbA1c test, and then referring these patients to a diabetes prevention program or self-management program. This initial ED/UC visit integrated with a referral and follow-up procedure may improve patient care access with minimal ED resource utilization. This study did not evaluate whether integrating such a screening program into the ED/UC system would potentially adversely affect patient flow or assess clinician adoption even with an EHR warning.

## LIMITATIONS

As a retrospective, risk-stratification study, several limitations should be noted. First, the data were collected from the EHR of a single healthcare system in a region of the country with a known high prevalence of the target disease, diabetes. Patients may have been members of another healthcare system that did not share data with the study location. The results obtained may not be generalizable to other geographic regions of the United States. Second, our classification and labeling of the patients was based on limited clinical, demographic, pharmaceutical, and laboratory information, with no confirmatory or fasting tests performed in a non-acute setting. Race and ethnicity were gleaned from the EHR that is generated upon patient registration and are self-reported. We did not use the current ADA standard glycemic values for diabetes; rather, we set the threshold higher due to the unscheduled acute setting. Additionally, it is recognized that hypoglycemic medications are used to treat diseases other than diabetes.

Finally, and as with any large dataset, missing and misaligned data points recorded in the EHR from multiple databases were not tested for bias. We focused our analysis only on the last known ED/UC visit, augmented with historical medical data, with 0.067% of the patients categorized in multiple classes and kept in the dataset.

## CONCLUSION

This proof-of-concept model shows the potential of incorporating clinical decision-making rules via advanced data analytics algorithms into the ED/UC EHR to identify an at-risk population for diabetes. The geographic information system mapping of EHR clinical data with other public datasets may further inform decision-makers of where and how interventions should be crafted to address this complex disease. The proposed preventive screening program may be most beneficial in areas where limited healthcare access exists, but where community healthcare agents are well established. This will ensure that the proposed follow-up mechanism of the referral from the ED/UC to a community-based diabetes program will be successful. Future work will need to address the development of a clinician-adoptable, real-time predictive model and evaluate patient post-visit resources required to improve the health of individuals and our community in a region of the country with a high prevalence of diabetes.

## Figures and Tables

**Table 1:** Demographic and classification characteristics of the emergency department (ED) / urgent care (UC) patients.

Variable	1 ED/UC visit/year	2 or 3 ED/UC visits/year	≥ 4 ED/UC visits/year	Total
Number of unique patients	173,364 (63.53)	78,054 (28.60)	21,469 (7.87)	272,887 (100)
Gender				
Male	80,457 (46.41)†	33,420 (42.82)	8,342 (38.86)	122,219 (44.79)‡
Female	92,873 (53.57)	44,629 (57.18)	13,126 (61.14)	150,628 (55.20)
Other	34 (0.02)	5 (<0.01)	1 (<0.01)	40 (0.01)
Race/Ethnicity				
White	129,557 (74.73)	58,517 (74.97)	15,715 (73.20)	203,789 (74.68)
African-American	29,948 (17.27)	15,098 (19.34)	4,918 (22.91)	49,964 (18.31)
Hispanic	8,900 (5.13)	2,894 (3.71)	517 (2.41)	12,311 (4.51)
Other§	4,959 (2.86)	1,545 (1.98)	319 (1.49)	6,823 (2.50)
Age (years)				
< 20	7,427 (4.28)	2,310 (2.96)	389 (1.81)	10,126 (3.71)
20–39	64,304 (37.09)	28,266 (36.21)	7,692 (35.83)	100,262 (36.74)
40–59	56,348 (32.50)	24,604 (31.52)	6,806 (31.70)	87,758 (32.16)
60–79	36,796 (21.22)	17,174 (22.00)	4,744 (22.10)	58,714 (21.52)
> 80	8,489 (4.90)	5,700 (7.30)	1,838 (8.56)	16,027 (5.87)
Insurance				
Medicare**	38,143 (22.00)	21,225 (27.19)	7,385 (34.40)	66,753 (24.46)
Medicaid††	13,038 (7.52)	8,288 (10.62)	3,963 (18.46)	25,289 (9.27)
Self-pay	441 (0.25)	230 (0.29)	90 (0.42)	761 (0.28)
Commercial‡‡	98,709 (56.94)	40,871 (52.36)	8,379 (39.03)	147,959 (54.22)
Unknown	23,033 (13.29)	7,440 (9.53)	1,652 (7.69)	32,125 (11.77)
Risk Classification				
(1) Otherwise “healthy”	92,744 (53.50)	36,432 (46.68)	8,014 (37.33)	137,190 (50.27)
(2) Labeled having prediabetes	529 (0.31)	597 (0.76)	304 (1.42)	1,430 (0.52)
(3) Labeled having diabetes	14,682 (8.47)	10,143 (12.99)	4,480 (20.87)	29,305 (10.74)
(3.1) Well managed	667 (0.38)	529 (0.68)	244 (1.14)	1,440 (0.53)
(3.2) Management challenged	13,442 (7.75)	9,183 (11.76)	4,027 (18.76)	26,652 (9.77)
(3.3) Poorly managed	705 (0.41)	580 (0.74)	274 (1.28)	1,559 (0.57)
(4) Unlabeled at-risk	65,452 (37.75)	30,963 (39.67)	8,732 (40.67)	105,147 (38.53)
(4.1) Unlabeled diabetes at-risk	8,349 (4.82)	3,303 (4.23)	853 (3.97)	12,505 (4.58)
(4.2) Unlabeled prediabetes at-risk	57,103 (32.94)	27,660 (35.44)	7,879 (36.70)	92,642 (33.95)

* Data are reported as n (%).

† % in columns 2, 3 and 4 are a calculated function of the total number of unique patients identified for each of the three subpopulations.

‡ % in column 5 are a calculated function of the total number of unique patients identified in the data set.

§ “Other” category includes American Indian or Alaska Native, Asian, biracial or multiracial, unknown, Native Hawaiian or other Pacific Islander, Patient refused, Other.

** Medicare and Medicare Advanced.

†† Medicaid, Medicaid managed care organization, and pending Medicaid.

‡‡ Commercial, Blue Cross, Liability, Managed Care, Tricare, Worker’s Comp, Other.

**Table 2:** Prevalence of labeled and unlabeled patients with diabetes as a function of race/ethnicity.

	Total	% Per race category§§	(3) % Labeled having diabetes***	(4.1) % Unlabeled diabetes at-risk	Total diabetes patients
Number of unique patients	272,887		29,305	12,505	41,810
White	203,789	74.68%	10.39%	4.75%	15.13%
African-American	49,964	18.31%	13.05%	5.20%	18.25%
Hispanic	12,311	4.51%	8.37%	5.15%	13.52%
Others	6,823	2.50%			

§§ % are calculated as a function of the total number of unique patients.

*** % calculated as a function of the race/ethnic categories of interest.
